# A heuristic algorithm solving the mutual-exclusivity-sorting problem

**DOI:** 10.1093/bioinformatics/btad016

**Published:** 2023-01-12

**Authors:** Alessandro Vinceti, Lucia Trastulla, Umberto Perron, Andrea Raiconi, Francesco Iorio

**Affiliations:** Computational Biology Research Centre, Human Technopole, 20157 Milano, Italy; Computational Biology Research Centre, Human Technopole, 20157 Milano, Italy; Computational Biology Research Centre, Human Technopole, 20157 Milano, Italy; Institute for Applied Mathematics “Mauro Picone”, National Research Council (IAC-CNR), 80131 Napoli, Italy; Computational Biology Research Centre, Human Technopole, 20157 Milano, Italy

## Abstract

**Motivation:**

Binary (or Boolean) matrices provide a common effective data representation adopted in several domains of computational biology, especially for investigating cancer and other human diseases. For instance, they are used to summarize genetic aberrations—copy number alterations or mutations—observed in cancer patient cohorts, effectively highlighting combinatorial relations among them. One of these is the tendency for two or more genes not to be co-mutated in the same sample or patient, i.e. a mutual-exclusivity trend. Exploiting this principle has allowed identifying new cancer driver protein-interaction networks and has been proposed to design effective combinatorial anti-cancer therapies rationally. Several tools exist to identify and statistically assess mutual-exclusive cancer-driver genomic events. However, these tools need to be equipped with robust/efficient methods to sort rows and columns of a binary matrix to visually highlight possible mutual-exclusivity trends.

**Results:**

Here, we formalize the mutual-exclusivity-sorting problem and present MutExMatSorting: an R package implementing a computationally efficient algorithm able to sort rows and columns of a binary matrix to highlight mutual-exclusivity patterns. Particularly, our algorithm minimizes the extent of collective vertical overlap between consecutive non-zero entries across rows while maximizing the number of adjacent non-zero entries in the same row. Here, we demonstrate that existing tools for mutual-exclusivity analysis are suboptimal according to these criteria and are outperformed by MutExMatSorting.

**Availability and implementation:**

https://github.com/AleVin1995/MutExMatSorting.

**Supplementary information:**

Supplementary data are available at *Bioinformatics* online.

## 1 Introduction

Large comprehensive biological datasets from multiple ‘omics approaches are being increasingly generated and released in the public domain (Costa, 2014; Deutsch *et al.*, 2019; Perez-Riverol *et al.*, 2019; Wörheide *et al.*, 2021). This offers computational biologists and bioinformaticians an unprecedented chance to gain mechanistic insights into cellular processes and biological phenomena involved in the aetiology of cancer and other human diseases (Alexandrov *et al.*, 2020; Golriz Khatami *et al.*, 2020; Nik-Zainal *et al.*, 2015).

For analytical or visualization purposes, datasets representing, for example, somatic mutations across extensive collections of cancer patients (Cancer Genome Atlas Research Network *et al.*, 2013; Zhang *et al.*, 2011) or genetic dependencies across large panels of cancer *in vitro* models (Behan *et al.*, 2019; Dempster *et al.*, 2019; Dwane *et al.*, 2021; Meyers *et al.*, 2017; Tsherniak *et al.*, 2017) can be modelled through bipartite networks or adjacency- and presence/absence matrices. These are binary (or Boolean) matrices whose entries can assume only one value in {TRUE, FALSE} or {0,1}, providing a practical, portable and easily visualizable data summary in a wide variety of fields, including bioinformatics. Notably, binary matrices (BMs) have been recently employed in computational cancer research for identifying and visualizing sets of genes with a tendency to be mutated or copy number amplified/deleted in a mutually exclusive manner across samples or patients (Cerami *et al.*, 2012). Recent works have shown that different cancers are characterized by heterogeneous genomic alterations, which tend to affect genes involved in a limited number of diverse biological processes. In addition, multiple genomic aberrations do not appear to occur in genes involved in the same biological process or pathway (Watson *et al.*, 2013). These observations are consistent with an elegant principle of evolutionary parsimony where plurality is not posited without necessity. In fact, it has been reported that most often, a single mutated node of a biological pathway is sufficient to make it constitutively active, providing selective advantages to cancer cells; thus, mutations in other genes/nodes do not tend to co-occur in the same pathway (El Tekle *et al.*, 2021).

In conclusion, novel sets of genes that are frequently mutated collectively across large cohorts of cancer patients but in a mutually exclusive fashion—i.e. never or rarely together in the same patient—might be part of the same oncogenic pathway. Consistently, identifying gene sets with this tendency has recently unveiled new oncogenic network modules (Ciriello *et al.*, 2012; Vandin *et al.*, 2012). More recently, sets of genes with a tendency to be vitally essential in a mutually exclusive fashion across panels of cancer *in vitro* models have revealed synthetic lethalities that could be targeted by combinatorial cancer therapies (Mullard, 2017; Srihari *et al.*, 2015). Methods based on these principles have also highlighted the need for new computationally efficient tools to properly simulate constrained null models for testing the significance of such mutual-exclusivity patterns in gene mutations/essentialities when analysing large datasets. To this aim, we had previously designed computationally efficient methods to simulate samples from the uniform distributions of BMs with prescribed marginal totals (Gobbi *et al.*, 2014). Subsequently, we have extended these approaches to the problem of randomizing signalling and pathway maps while preserving the functional characterization of individual nodes (Iorio *et al.*, 2016b).

Here, we tackle another computationally hard problem involving the manipulation of BMs to highlight possible row-wise mutual-exclusivity trends across patterns of non-zero entries (Johnson *et al.*, 2004), i.e. mutual-exclusivity sorting. We have designed a computationally efficient heuristic algorithm to solve this problem and implemented it as a general-purpose and user-friendly R package: MutExMatSorting. Our package provides a fast and convenient solution to rearrange a binary matrix (BM) to visually highlight patterns of mutual exclusivity across genes or cancer functional events (i.e. rows). To demonstrate the scalability and accuracy of our package, we also present its performances and computational time requirements obtained when applied to BMs of different sizes and densities of non-zero entries compared to other state-of-the-art tools.

## 2 Materials and methods

### 2.1 A heuristic algorithm solving the mutual-exclusivity-sorting problem

**Fig. 1.**
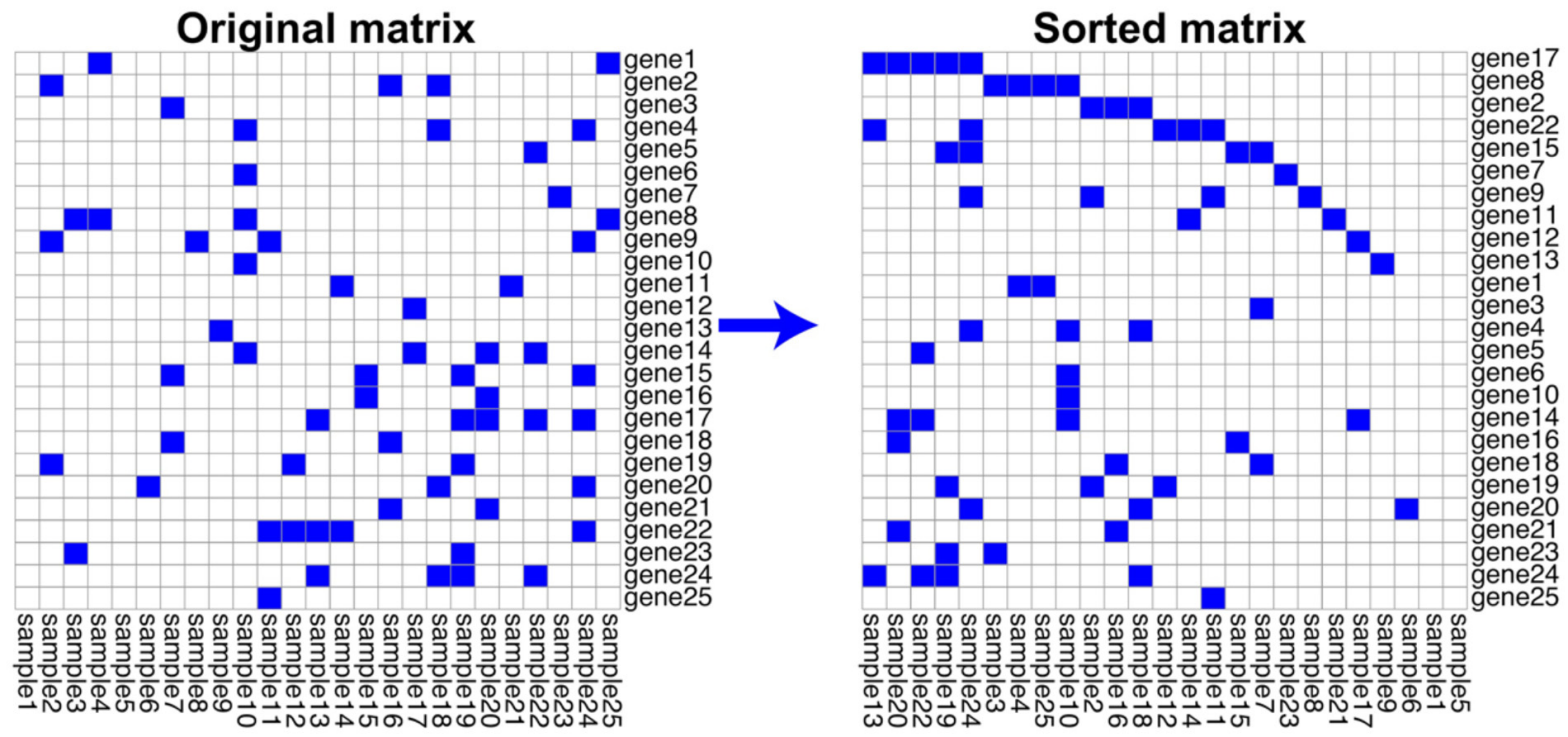
Example of mutual-exclusivity sorting performed by MutExMatSorting on a BM. By defining a run as a sequence of consecutive non-zero entries in a given row of a matrix, MutExMatSorting reorders rows and columns of that matrix to minimize overlapping runs across rows and maximize the runs’ length. This highlights possible mutual-exclusivity trends across rows

We have designed MutExMatSorting: a heuristic algorithm that solves the *mutual-exclusivity-sorting* problem for a BM (Fig. 1). For simplicity, we stick to the example of a BM summarizing genomic alterations across a cohort of cancer patients, which we defined in the previous section. Thus, we will use *genes* and *samples* to refer to rows and columns of the BM, respectively.

Briefly, a group of genes will be mutually exclusively mutated in such a matrix if they do not tend to have sequences of non-zero entries that overlap vertically or, in other words, they are not co-mutated in the same sample. More formally, a run within a row of a BM is defined as a sequence of consecutive non-zero entries (Johnson *et al.*, 2004). Sorting rows and columns of a BM to minimize the number of vertically overlapping runs is an effective method to visually highlight possible patterns of mutually exclusively mutated genes, thus patterns of mutually exclusive non-zero entries across different rows. Finding the absolute minimum of this overlap has been characterized as an NP-hard problem (Johnson *et al.*, 2004). Here, we refer to this problem as the *mutual-exclusivity-sorting* of a BM.

To solve this problem, MutExMatSorting starts by labelling each gene and sample represented in the input BM **B** as ‘uncovered’ and by initializing two empty vectors, respectively, for the set of ‘covered’ genes G* and the set of ‘covered’ samples S*. Following this step, a series of iterations start until there are no uncovered genes (indicated by **G**) and uncovered samples (**S**) left. In each iteration, the ‘best-in-class gene’ g* is determined. This is the uncovered gene g with the maximal exclusive coverage, defined as the number of uncovered samples in which *g* is mutated (**B**_*g,s*_) minus the number of uncovered samples in which any other gene *g′* is mutated (**B**_*g′,s*_):

$$g^{*}=\underset{g\in G}{\arg\max}\{\sum_{s\in S}(B_{g,s}-\sum_{q\prime \in G,q\prime \neq q}B_{g\prime ,s})\}$$

The gene g* is then labelled as covered and appended to the vector G*. Finally, the samples hosting mutations in g* (i.e. non-zero entries) are also labelled as covered and added to the S* vector.

The iterations continue until there are no more uncovered genes or no more uncovered samples left. In the latter case, the remaining uncovered genes are added to G*.

Then, the algorithm initializes an empty vector of samples **L** and a vector of genes **G′** = G*, and all the samples are again labelled as uncovered, i.e. **S **= S* and S* = ∅. Then, for each gene *g*, in **G′** (considered in the same order in which genes appear in this vector) and as long as there are uncovered samples, the uncovered samples hosting mutations in *g* are sorted in decreasing order according to their *exclusive coverage* with respect to *g*, defined as:

$$L_{s}=B_{g,s}[1-\sum_{q\prime \in G\prime ,q\neq q}B_{g\prime s}].$$

They are attached in the resulting order to vector **L** and labelled as covered. This final step allows sorting the columns to maximize the length of the runs (i.e. the consecutive number of non-zero entries in a row). Finally, genes and patients in the input BM **B** are rearranged to reflect the order in which they appear, respectively, in G* and **L**.

A first, less efficient version of this algorithm with no dedicated software package was introduced in Iorio *et al.* (2018). A pseudocode of the MutExMatSorting algorithm is provided in the Supplementary data.

### 2.2 Time complexity analysis

We separately analyse the computational complexity of each of the two steps of our algorithm: the first one in which the G* vector is constructed and the second one that assembles the **L** vector.

In what follows, let *n* be the number of rows (i.e. genes), and *m* the number of columns (i.e. samples) of the input BM. In each iteration of the first step, the algorithm computes the *maximal exclusive coverage* score for each uncovered gene to identify the best-in-class one. For each uncovered gene g, this requires cycling through each uncovered sample to check whether g is mutated in that sample and how many other uncovered genes are also mutated in the same sample. For each sample, these checks can be performed in constant time, assuming we know in advance the column-wise marginal totals of BM.

The incumbent best-in-class gene g* is saved along this process. Therefore, identifying the best-in-class gene in each iteration has a complexity of O(*nm*). After identifying g*, the algorithm iterates again through all samples in O(*m*) to label as covered those in which g* is mutated. Overall, the computational complexity of each iteration is once again O(*nm*). Since the procedure we just described is iterated until all genes are covered, the complexity of the first part of the algorithm is O(*n^2^m*).

Let us now focus on the second part. In each iteration, for each gene *g* taken from **G′**, the algorithm computes the maximal exclusive coverage score with respect to g associated with the uncovered samples in which g is mutated. Similarly to what is described above, these computations are performed in O(*m*). The algorithm then sorts the scores associated with these samples in O(*m* log *m*). Again, it scrolls the samples to mark the newly covered ones as such in O(*m*). The complexity of each iteration is O(*m* log *m*).

Finally, since these computations are iterated over all the elements of **G′**, the complexity of the second part is O(*nm* log *m*). In conclusion, the final complexity is O(*n*^2^*m* + *nm* log *m*).

### 2.3 MutExMatSorting R package

The MutExMatSorting R package is publicly available and fully documented at https://github.com/AleVin1995/MutExMatSorting. It encompasses five core functions, three wrapped in the main *MExMaS.HeuristicMutExSorting* function implementing our heuristic algorithm. The two other functions are wrapped in the *MExMaS.MEMo* function, implementing the matrix-sorting procedure included in MEMo (Ciriello *et al.*, 2013): a software for identifying gene modules whose alterations tend to show mutual-exclusivity patterns, which we consider here for comparison purposes.

Our package takes in input a BM with unique rows and column identifiers or a matrix with unnamed rows/columns (which will be named using row/column positions as default identifiers).

MutExMatSorting calls iteratively the function *MExMaS.findBestInClass*, which finds a best-in-class gene (defined in the previous section), and *MExMaS.rearrangeMatrix*, which rearranges the columns of the input matrix according to their exclusive coverage (also defined in the previous section). Finally, *MExMaS.MEMo* calls the *MExMaS.scoreCol* function internally to finalize the implemented rearrangements of the input BM.

### 2.4 Hardware and software used for the performance assessment

We executed performance assessment and comparative analyses of our algorithm locally on a macOS laptop with a 2.3 GHz Quad-Core Intel Core i7 processor and 16 GB 3733 MHz LPDDR4X memory. The operating system was Monterey v12.2.1. We used the RStudio v1.4.1717 with R v4.1.1 within an x86_64-apple-darwin17.0 platform.

The comparison between MutExMatSorting and the MEMo algorithm was carried out on a remote VMware machine running on CentOS 8.4 distribution due to the long-running time of the analysis. We used the R programming language v4.1.1 within an x86_64-conda-linux-gnu platform.

## 3 Results

In the first section, we provide a use case of the MutExMatSorting package to visually highlight patterns of mutual exclusivity using real-world data (see Section 3.1). Next, we assess the performance of our tool with respect to different values of matrix size and density (see Section 3.2). In Section 3.3, the performances of MutExMatSorting are compared to another matrix-sorting algorithm, namely MEMo (Ciriello *et al.*, 2013), and random sorting. In more detail, we first validate their performances on synthetic data using our mutual-exclusivity score (see Section 3.3.1) and an independent score (see Section 3.3.2). Finally, we compare them on a human cancer dependency dataset (see Section 3.3.3).

### 3.1 MutExMatSorting outcome on real cancer datasets

**Fig. 2.**
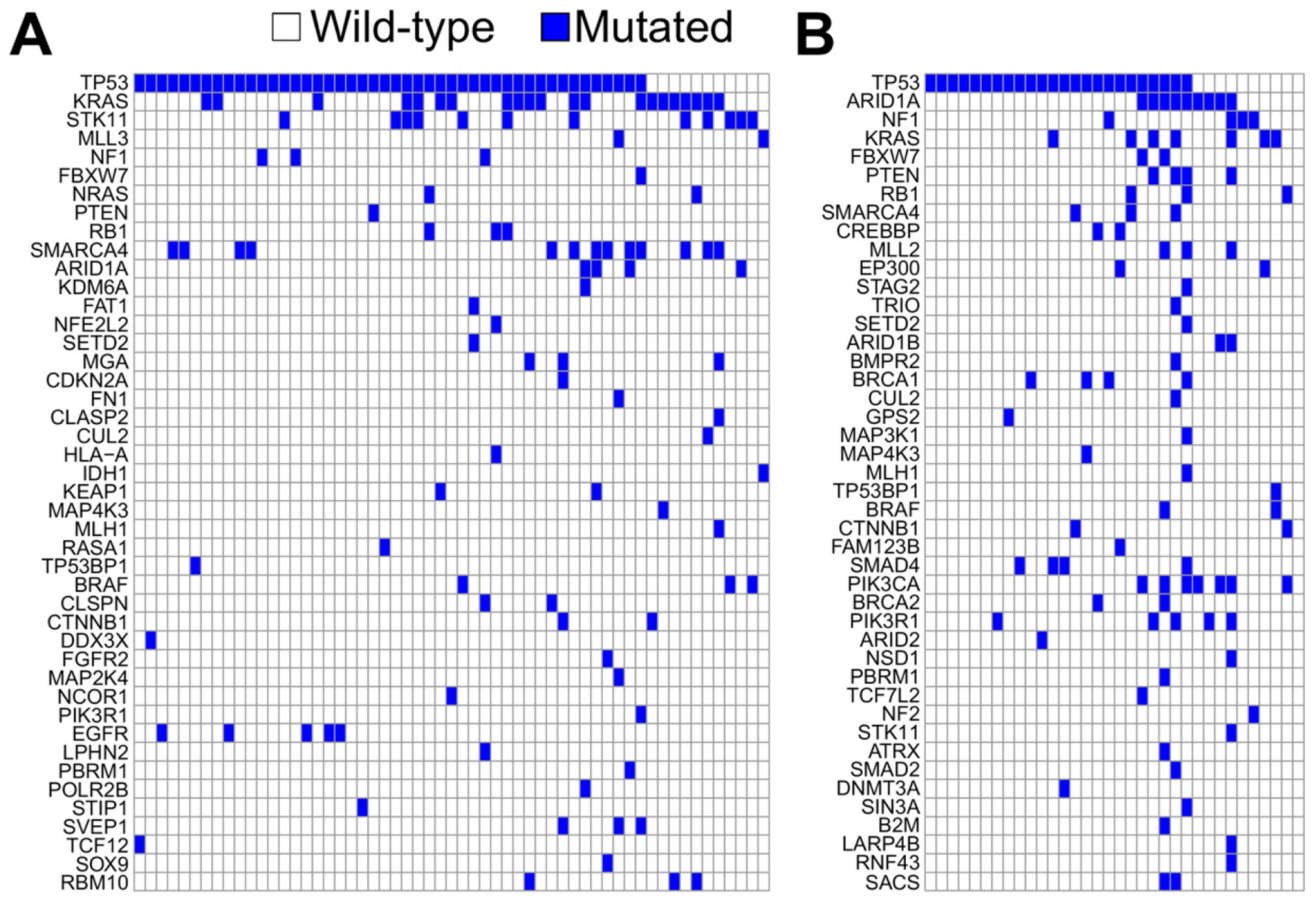
Mutual-exclusivity sorting performed by MutExMatSorting on cancer datasets. Visualization of MutExMatSorting outcome on binary matrices representing somatic mutations across cancer-driver genes in cell lines derived from lung adenocarcinoma (**A**) and ovarian (**B**) primary tumours

To visually highlight the rearranging capabilities of MutExMatSorting, we applied it to two binary matrices from Iorio *et al.* (2016a). These summarize somatic mutations in cancer-driver genes identified in lung adenocarcinoma (LUAD) and ovarian (OV) primary tumours, mapped onto cell line cohorts from matching tissues (respectively Fig. 2A and B).

The output obtained by processing the LUAD cell lines’ matrix (Fig. 2A) highlighted a possible mutual-exclusivity trend between TP53, KRAS and STK11.

It has been demonstrated (La Fleur *et al.*, 2019) that co-mutations in TP53 or STK11 confer poor prognosis in KRAS-positive patients. Indeed, TP53 mutations enhance signatures related to cell proliferation, whereas STK11 mutations suppress signatures of immune function (Schabath *et al.*, 2016). Thus, the negative prognostic outcome of co-mutations in TP53 or STK11 might be illustrated from a functional perspective by the proliferative drive and poor immune function in the two subgroups, respectively.

On the other hand, applying our method to the OV cell lines’ matrix (Fig. 2B) placed TP53 and ARID1A genes at the top, highlighting a possible mutual-exclusivity trend among these genes. Interestingly, several studies (Allo *et al.*, 2014; Reske *et al.*, 2021; Wu *et al.*, 2017; Xu and Tang, 2021) have shown that TP53 and ARID1A are frequently mutated across cancer samples but rarely in the same primary tumour. It has been shown that, especially in OV cancer cell lines, ARID1A directly interacts with TP53 (Guan *et al.*, 2011) to regulate the transcription of its targeted genes, such as p21, which can lead to subsequent cell cycle arrest. Following a parsimony principle, mutation of either gene is sufficient to turn off the tumour suppressor activity of the set of genes co-regulated by TP53 and ARID1A.

These examples demonstrate that MutExMatSorting can effectively visually highlight possible mutual-exclusivity trends between patterns of mutations when applied to real cancer genomics data.

Furthermore, we note that in a scenario where tens of thousands of genes are considered, using MutExMatSorting to visualize the whole rearranged matrix might be cumbersome. In this case, the user might be interested in applying MutExMatSorting to highlight patterns of mutual exclusivity and then representing a rearranged subset of the original matrix which contains said patterns (e.g. visualization of the top 25 rows).

### 3.2 Performance assessment on a typical desktop architecture

**Fig. 3.**
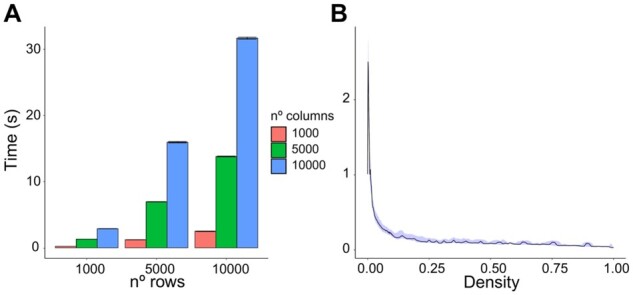
MutExMatSorting performances on binary matrices (BMs) of different sizes and densities. (**A**) Performance assessment when varying number of rows and columns in the BM. We kept the density (defined as the ratio between the number of non-zero entries over the total) equal to 0.1 across all instances. The error bar shows the 95% confidence interval obtained on 1000 iterations for every combination parameter. (**B**) Performance assessment when varying the density of non-zero entries in a BM of constant size (1000 × 1000). The blue shade is the 95% confidence interval obtained on 1000 iterations for every density instance we tested

We sought to assess the running time performances of our package across input binary matrices (BMs) of different sizes and different densities of non-null entries (Fig. 3) on a typical laptop (as described in Section 2).

First, we tested the impact of the BM size in terms of varying numbers of rows and columns (Fig. 3A) while keeping its density at a constant value of 0.1 (i.e. the non-zero entries make up 10% of the BM). We performed 1000 instance executions of our algorithm for each combination of parameters, using randomly assembled BM as input in each. Even for large matrices (e.g. 10 000 × 10 000), the running time of MutExMatSorting was under a minute.

On the other hand, the execution time varies more when assessed across different density values at a fixed matrix size (Fig. 3B). We tested different ranges of density from 10^−3^ to 1 on a BM of size 1000 × 1000 and, again, performed 1000 iterations for each instance. In particular, we tested the range of values from 10^−3^ to 10^−1^ with a 10^−3^ step increase and the remaining range of values with a 10^−1^ step increase. We observe a peak around the density of 2 × 10^−3^ in the execution time.

This is consistent with the improbable worst-case scenario represented by a shuffled diagonal matrix in which there is only one non-overlapping entry per row. In this case, only one row and one column are removed (i.e. considered as covered) in each best-in-class gene-seeking iteration.

### 3.3 Comparison with the MEMo sorting procedure

#### 3.3.1 Mutual-exclusivity coverage across different ranges of matrix size and density

We compared MutExMatSorting to a matrix-sorting procedure implemented in a state-of-the-art tool for identifying mutual-exclusivity patterns in cancer genomics datasets: Mutual Exclusivity Modules in Cancer (MEMo) (Ciriello *et al.*, 2013). MEMo identifies sets of genes with genomic alterations that tend not to co-occur in the same biological process and also implements a matrix-sorting method used for visualization purposes only. This procedure first sorts the rows of the input BM in decreasing order according to the number of non-zero entries. Then, it performs a column-wise sorting based on an exponentially weighted sum score, where non-zero entries at the top of the matrix are assigned larger scores than those found at the bottom.

This is easily achieved by considering, for example, the pattern of 1/0 entries in each column as numbers expressed in binary notation.

As a comparison criterion, we considered for each sorted BM outputted by each method an overall mutual-exclusivity coverage score, computed as follows. For a given BM, and each gene iteratively considered in the same order as they appear in the BM’s rows, we computed the mutual-exclusivity coverage for that gene (as defined in Section 2), then we removed the gene and the samples in which that gene was mutated from the BM. Summing the resulting gene-wise mutual-exclusivity coverage scores yield the overall BM one.

**Fig. 4.**
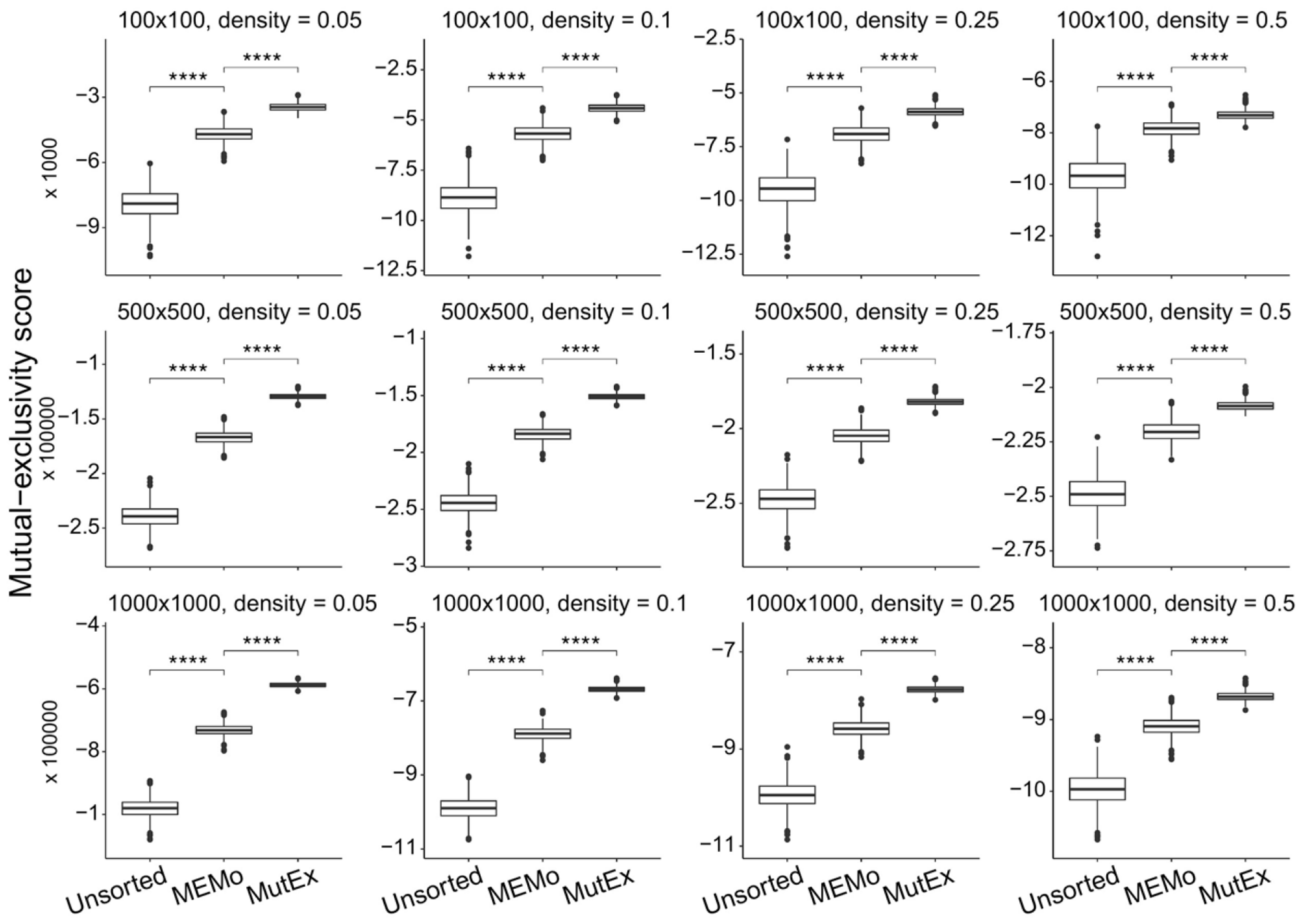
Comparison between MutExMatSorting and MEMo algorithms in maximizing the mutual-exclusivity coverage score. We compared the MutExMatSorting and MEMo algorithms in their ability to rearrange an input BM for highlighting mutual-exclusivity patterns. In particular, we tested different matrix sizes (i.e. the number of rows and columns) and densities (i.e. the ratio between the non-zero entries and the total entries in the BM). For each combination of parameters, we performed 1000 iterations. As a baseline, we considered the original unsorted BM. All differences, in terms of performances, between the unsorted and MEMo-arranged matrix as well as those between the latter and MutExMatSorting-arranged matrix are highly significant (*P* < 10^−4^)

We calculated this score on three types of BMs: the original unsorted BM, the MEMo-arranged BM and the MutExMatSorting-arranged BM. We compared the two algorithms (Fig. 4) using different settings of matrix size (i.e. 100, 500 and 10 000 rows and columns) and density (i.e. 0.05, 0.1, 0.25 and 0.5) and performed 1000 iterations for each combination of parameters. Due to the long-running time, we executed this analysis on a remote virtual machine (see Section 2).

All of the comparisons yielded highly significant (*P* < 10^−4^) differences, with MutExMatSorting-arranged BMs showing significantly larger scores than MEMo-arranged BMs. In addition, the range of scores outputted by MutExMatSorting tends to have a lower variance compared to those of the other input BMs.

#### 3.3.2 Capability of highlighting mutual-exclusivity trends on synthetic data

**Fig. 5.**
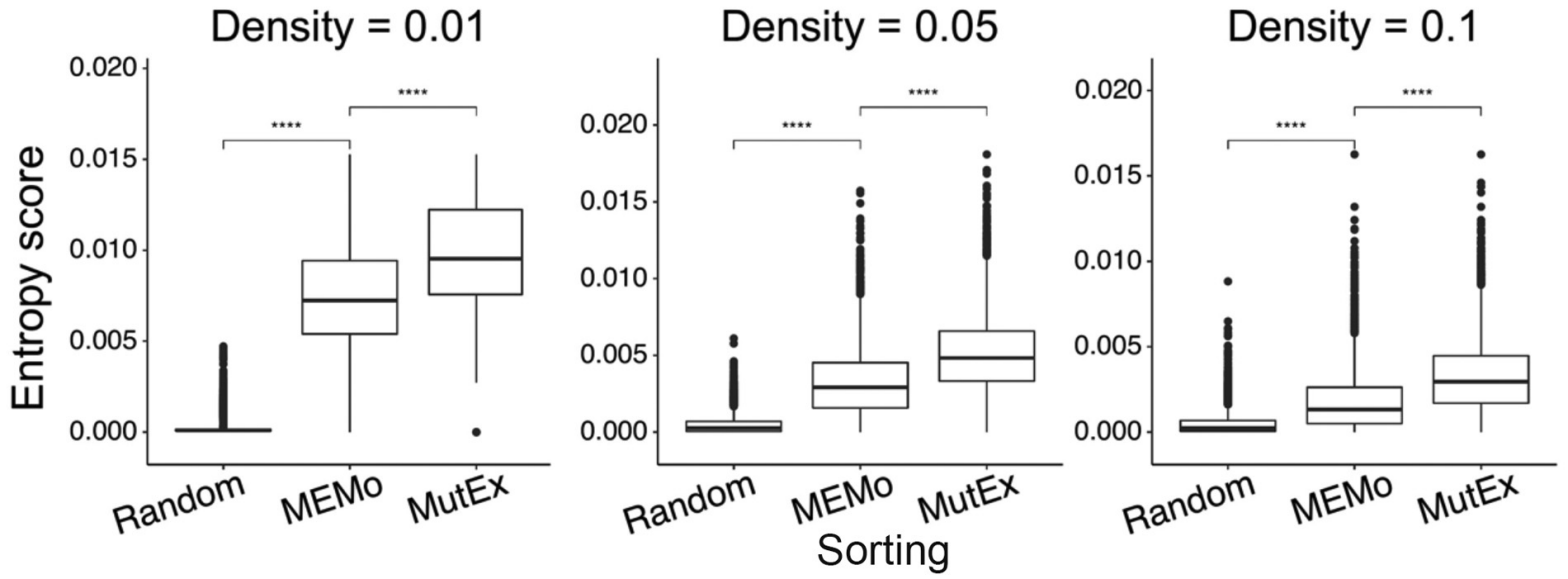
Comparison between MutExMatSorting and MEMo algorithms for the identification of mutual-exclusivity patterns using an entropy-based score. We compared the MutExMatSorting and MEMo algorithms in their ability to rearrange an input BM of fixed size (1000 × 1000) with known patterns of mutual exclusivity and co-occurrent binary events. In particular, we introduced a set of 10 genes with highly mutually exclusive mutations (i.e. no overlap of non-zero entries) and another set of 10 genes with a high degree of co-occurrent mutations. We tested different matrix densities (i.e. the ratio between the non-zero entries and the total entries in the BM) and for each combination of parameters, we performed 1000 iterations. As a baseline, we considered a random sorting strategy where the BM is randomly shuffled row wise. All differences, in terms of performances, between the randomly sorted and MEMo-arranged matrix as well as those between the latter and MutExMatSorting-arranged matrix are highly significant (*P* < 10^−4^) based on an entropy score

Using an independent performance metric, we further compared MutExMatSorting and MEMo on synthetic data with controlled mutual-exclusivity trends. To this aim, we considered a matrix of fixed size (i.e. 1000 genes × 1000 samples) injected with sets of mutually exclusive or co-occurrent mutations and compared MutExMatSorting and MEMo sorting procedures using an entropy-based score implemented in the infotheo package (Cover and Thomas, 1991; Meyer, 2008) applied to their outputted matrices (Fig. 5). While building the synthetic matrix, we introduced a set of 10 genes whose mutations are highly mutually exclusive (i.e. no overlap between non-zero entries), a set of 10 genes with largely overlapping mutations across samples, and other genes containing random mutations at different densities (i.e. 0.01, 0.05 and 0.1).

For each density value, we computed an entropy score for all possible pairs of genes appearing in the top 10 rows of the matrices outputted by MutExMatSorting and MEMo, using the infotheo package across 1000 replicates. If a sorting strategy can correctly detect and rank the highly mutually exclusive gene pairs, it will result in larger entropy scores. Indeed, in all comparisons between MutExMatSorting and MEMo, as well as MEMo and a random shuffling control, we obtained highly significant (*P* < 10^−4^) improvements in entropy scores, with MutExMatSorting-arranged BMs showing larger scores than MEMo-arranged BMs. This indicates that our MutExMatSorting outperforms MEMo even when using an independent metric on synthetic data mimicking typical cancer mutational signatures.

#### 3.3.3 Capability of highlighting mutual-exclusivity trends on real data

**Fig. 6.**
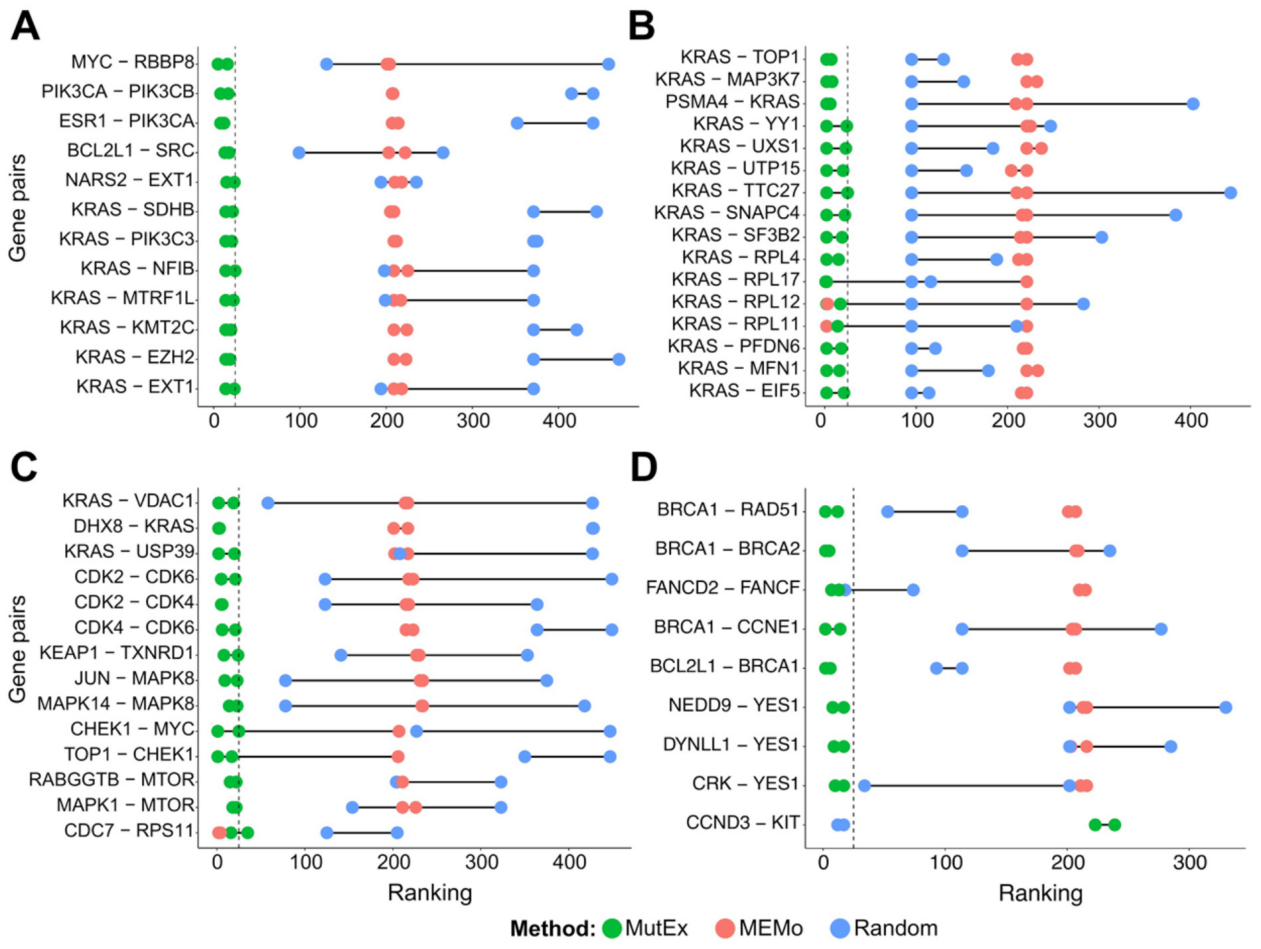
Comparison between MutExMatSorting, MEMo and random strategies in highlighting known human synthetic lethal pairs across different cancer types. Row-based ranking of known cancer-specific synthetic lethal (SL) pairs in breast carcinoma (**A**), colorectal carcinoma (**B**), lung adenocarcinoma (**C**) and ovarian carcinoma (**D**) upon MutExMatSorting, MEMo or random sorting of the binary dependency dataset derived from the Project Score. Only SL pairs with a confidence score > 0.4 were selected. For each cancer type, we subset the binary dataset considering the corresponding cancer cell lines and genes part of the SL pairs. We also added 200 core-fitness and 200 never-essential genes as negative controls (i.e. non-mutually exclusive). The figures show only the SL pairs found in the top 25 rows (dashed line) of at least one of the sorted matrices

As highlighted in Ciriello *et al.* (2012), patterns of mutual exclusivity in cancer arise from a scenario where the alteration of a second gene either (i) does not offer any further selective advantage or (ii) leads to a significant decrease in cell viability, also known as synthetic lethality (SL) (O’Neil *et al.*, 2017). To extend the comparison between the MutExMatSoring and MEMo algorithms, we compared their ability to recapitulate known SL gene pairs (Fig. 6) derived from the SynLethDBv2 database (Wang *et al.*, 2022) on a binary dependency dataset derived from Project Score (Dwane *et al.*, 2021). In this dataset, a non-zero entry in the position [*i, j*] corresponds to an observed reduction of viability of the cell line *j*, upon CRISPR-cas9 knock-out of gene *i*, i.e. gene *i* is vitally essential for cell line *j*.

We considered known human SL pairs (Wang *et al.*, 2022) with a quantitative score greater than 0.4 across four cancer types: breast carcinoma, colorectal carcinoma, LUAD and OV carcinoma. The confidence score of an SL pair is given by a weighted aggregation of different sources of evidence (e.g. CRISPRi, RNAi or drug inhibition screenings). For each cancer type, we subset the Project Score dataset by considering only the corresponding cell lines and the genes which are part of each SL pair. In addition, we included 200 core-fitness genes (i.e. genes essential in at least 90% of the cell lines in the cancer type) and 200 never-essential genes (i.e. not essential in any cell line) as negative controls.

We checked how many SL pairs were placed in the top 25 rows of at least one of the sorted matrices. For most cases, we found SL gene pairs at the top of the matrices sorted with MutExMatSorting, especially for breast carcinoma (Fig. 6A) and colorectal carcinoma (Fig. 6B).

Compared to MutExMatSorting, MEMo could better recapitulate only one SL pair in LUAD (CDC7–RPS11, Fig. 6C). A random strategy performed better only in one SL pair instance in OV carcinoma (CCND3–KIT, Fig. 6D).

## 4 Discussion

A key challenge in cancer genomic research consists in elucidating possible interplays between various genetic aberrations (e.g. somatic mutations, copy number variations, methylations etc.). Indeed, alterations that tend not to occur in the same pathway may highlight either evolutionary parsimony (i.e. further mutations do not confer any additional selective advantage) or SL, a phenomenon where the cell is able to tolerate a single genetic disruption, whereas the occurrence of multiple disruptions is not tolerated lead to cell death. The latter is of particular relevance for the development of combinatorial therapies and many studies have already shown the potential of SL as a therapeutic strategy in cancer treatment (Behan *et al.*, 2019; Helming *et al.*, 2014; Hoffman *et al.*, 2014; Lord and Ashworth, 2017).

We designed a heuristic algorithm implemented in the R package MutExMatSorting, rearranging rows and columns of a BM in a way that highlights possible trends of mutual-exclusivity patterns. Additionally, matrix reordering is an NP-hard problem that has many implications in computer science. The most well-known example in bioinformatics is the Burrows–Wheeler transform (Burrows and Wheeler, 1994): a lossless data compression algorithm that applies a reversible transformation to reorganize text. Therefore, implementing heuristic solutions that tackle this task is an essential field of study. Our algorithm sorts a sparse BM in a time-efficient manner, and since it also maximizes the length of runs across rows, the rearranged matrix is easier to compress.

We have tested the package to ensure the correctness of obtained results and the accuracy and speed with binary matrices of different sizes and densities. We observed the algorithm scales well as row and column size increase in terms of execution time. Although the execution time increases significantly when approaching the worst-case scenario for a fixed matrix size, we notice that the very low density of such matrices makes them unlikely to find any practical application as it decreases linearly as the matrix scales up.

We also performed a robust benchmarking between MutExMatSorting and MEMo, another popular method to visually highlight the occurrence of genomic alterations across samples. We tested these two methods, together with a random sorting strategy, both on synthetic and real-world data. For the comparison based on synthetic data, we applied two different metrics: mutual-exclusive coverage, which corresponds to the score our method optimizes during the sorting procedure, and an orthogonal entropy-based score implemented in the infotheo R package. In both instances, rows (i.e. genes) found at the top of the MutExMatSorting-rearranged matrices showed a significantly larger mutual exclusivity compared to MEMo and random sorting. For the comparison based on real-world data, instead, we showed that MutExMatSorting is better able to recapitulate known synthetic lethal gene pairs on the binary dependency matrix derived from Project Score across different cancer types.

Future developments of our package might include integrating our method with CELLector (Najgebauer *et al.*, 2020), a recently published tool for patient-genomics-guided selection of *in vitro* cancer cell lines and for the identification of hierarchical co-occurrence of cancer functional events (i.e. somatic mutations, copy number alterations and hyper-methylations). These tools could be combined to highlight more complex patterns such as mutually exclusive sets of co-mutated genomic alterations. In particular, the first run of CELLector could identify co-occurrent mutational signatures. These could be aggregated as ‘meta-genes’, and a following run of MutExMatSorting would then identify mutual-exclusivity patterns.

In conclusion, MutExMatSorting is an easy-to-use tool, written in R, aiming at unravelling possible *de novo* mutually exclusive patterns, based only on the provided genomic data. This has important applications in bioinformatics as well as other computational domains like data compression.

## Supplementary Material

btad016_Supplementary_Data

## Data Availability

MutExMatSorting is available at the following GitHub repository: https://github.com/AleVin1995/MutExMatSorting. Binary matrices summarizing somatic mutations (see Section 3.1) from (Iorio *et al.*, 2016a) can be downloaded from the Genomics of Drug Sensitivity in Cancer (GDSC) database (https://www.cancerrxgene.org/gdsc1000/GDSC1000_WebResources/Home.html) or can be derived from the CELLector R package (https://github.com/francescojm/CELLector). The Project Score dataset (see Section 3.3.3) can be downloaded from the Project Score web portal (https://score.depmap.sanger.ac.uk/). Human synthetic lethal pairs (see Section 3.3.3) are available at the SynLethDBv2 database (https://synlethdb.sist.shanghaitech.edu.cn/v2/).
