# Supplementary material for: A heuristic algorithm solving the mutual-exclusivity-sorting problem
Source: Bioinformatics. 2023 Jan 12;39(1):btad016. doi: 10.1093/bioinformatics/btad016 (PMC9857977; doi:10.1093/bioinformatics/btad016)
Supplement: btad016_Supplementary_Data [file btad016_supplementary_data.pdf]

Algorithm Heuristic Mutual Exclusivity Sorting

Input: BM: *nxm* binary matrix
Output: BM': Sorted *nxm* binary matrix

```
Procedure    HeuristicMutExSorting(BM)
    uncoveredGenes = 1:n
    uncoveredSamples = 1:m

    while uncoveredGenes > 0 AND uncoveredSamples > 0
        bm = BM[uncoveredGenes, uncoveredSamples]
        bestInClass = findBestInClass(bm)

        if uncoveredGenes > 1 then
            sortedRows = bestInClass
        else
            sortedRows = concatenate(sortedRows, bestInClass)
        end if

        uncoveredGenes = diff(uncoveredGenes, sortedRows)
        uncoveredSamples = diff(uncoveredSamples, columns(bm[bestInClass,] != 0))
    end while

    sortedRows = concatenate(sortedRows, uncoveredGenes)
    sortedColumns = rearrangeMatrix(B, sortedRows)
    BM' = BM[sortedRows, sortedColumns]

    return BM'
end procedure


Procedure    findBestInClass(bm)
    exclusiveCoverage = sum(2 · bm – columnSum(bm))
    g = argmax g ∈ rows(bm) (exclusiveCoverage)

    return g
end procedure


Procedure    rearrangeMatrix(BM, sortedRows)
    remainingSamples = 1:m
    columnOrder = init vector

    for g in sortedRows
        samples = sort.decreasing(2 · BM[g,] – columnSum(BM))
        columnOrder = concatenate(columnOrder, columns(BM[g, samples]!=0))
        remainingSamples = diff(remainingSamples, columnOrder)
    end for

    return columnOrder
end procedure
```
